# Longevity pathways and memory aging

**DOI:** 10.3389/fgene.2014.00155

**Published:** 2014-06-04

**Authors:** Ilias Gkikas, Dionysia Petratou, Nektarios Tavernarakis

**Affiliations:** ^1^Institute of Molecular Biology and Biotechnology, Foundation for Research and Technology-Hellas, HeraklionCrete, Greece; ^2^Department of Basic Sciences, Faculty of Medicine, University of Crete, HeraklionCrete, Greece

**Keywords:** Alzheimer’s disease, autophagy, dietary restriction, insulin/IGF-1 signaling, learning, mitochondria, neurodegeneration, TOR signaling

## Abstract

The aging process has been associated with numerous pathologies at the cellular, tissue, and organ level. Decline or loss of brain functions, including learning and memory, is one of the most devastating and feared aspects of aging. Learning and memory are fundamental processes by which animals adjust to environmental changes, evaluate various sensory signals based on context and experience, and make decisions to generate adaptive behaviors. Age-related memory impairment is an important phenotype of brain aging. Understanding the molecular mechanisms underlying age-related memory impairment is crucial for the development of therapeutic strategies that may eventually lead to the development of drugs to combat memory loss. Studies in invertebrate animal models have taught us much about the physiology of aging and its effects on learning and memory. In this review we survey recent progress relevant to conserved molecular pathways implicated in both aging and memory formation and consolidation.

## INTRODUCTION

During the past century, age-related memory impairments have emerged as one of the top public health threats. Both psychiatric and neurodegenerative disorders comprising schizophrenia, depression, Alzheimer’s disease (AD), Parkinson’s disease (PD), and Huntington’s disease (HD) are associated with age-related memory impairment. In humans, cognitive decline starts in mid-life and deepens with advancing age suggesting that the greatest risk factor is age itself. Thus, ultimately, prevention of these pathologies necessitates thorough understanding of the molecular mechanisms underlying their links with the aging process (Bishop et al., 2010).

Our knowledge of the molecular regulatory mechanisms of aging impinging on cognitive capacity is steadily increasing in recent years. Interestingly, analyses of vertebrate and invertebrate model systems suggest that molecular and genetic pathways regulating cognitive aging are highly conserved in yeast, flies, worms, and mammals (Barco et al., 2006; Ardiel and Rankin, 2010; Bishop et al., 2010; Kauffman et al., 2010). Accumulating evidence from these models suggest a dynamic association between cognitive functions and aging. Similarly to several phenotypes and biomarkers of aging, which can vary substantially among individuals, cognitive decline displays significant severity fluctuation within a population. Consequently, it is important to identify key regulators of both cognitive impairment and longevity pathways. A plethora of molecular and cellular studies indicate a strong entanglement between lifespan regulation pathways and cognitive decline or neurodegeneration. In this review, we survey the molecular mechanisms and genes associated with longevity that have also been implicated in cognitive aging (summarized in **Table 1**). We further focus on recent work in invertebrate model organisms linking learning and memory impairment with age.

**Table 1:** Conserved signaling pathway genes and cognitive aging in worms and flies.

Pathway	Gene	Cognitive aging effect	
		*Caenorhabditis elegans*	*Drosophila melanogaster*
Insulin signaling	*ins-1/INS*	Regulates gustatory associative learning, thermotaxis, and chemotaxis learning	NA^1^
	*daf-2/IGFR*	Inhibits growth cone initiation, axon growth and neuronal regrowth; loss-of-function improves thermotaxis associative learning and blocks chemotaxis/sensory integration learning	NA
	*age-1/PI3K*	Mutations improve thermotaxis learning with age but cause defects in chemotaxis associative learning	NA
	*daf-18/PTEN*	Loss-of-function decreases chemotaxis, odorant associative, and sensory integration learning	Inhibits axon regeneration
	*daf-16/FoXO*	Neuroprotective, promotes regeneration and neuronal migration; loss-of-function reduces associative and sensory integration learning	NA
Dietary restriction	*eat-2/DR*	Loss-of-function increases temperature–food association and impairs LTM^2^	DR^3^ decreases STM^4^ at mid-age, enhances MTM^5^ at young-age
TOR signaling	*rheb*	NA	Overexpression induces morphology defects, and decreases odor-sucrose MTM
	*rictor*	NA	Deficiency blocks LTM
Autophagy	*cdk-5*	NA	Olfactory learning and memory defects
	*apl-1/APPL*	Olfactory and gustatory learning defects, habituation delay	NA
	*unc-51/atg-1*	NA	Influence axonal and dendritic development affecting olfactory learning
Mitochondria	*sod-1*	NA	Memory impairment associated with less synapses and mitochondrial dysfunction
	*ucp-4, ucp-2*	Promote neuronal toxicity in Huntington’s disease models	Susceptible to Parkinson’s and Huntington’s diseases
	*ced-9/Debcl*	NA	Ameliorate cognition
	*clk-1*	Developmental and behavioral defects	Influence rhythmic behaviors

1 No information available, ^2^Long-term memory, ^3^Dietary restriction, ^4^Short-term memory, ^5^Mid-term memory.

## REDUCED INSULIN/IGF-1 SIGNALING PROMOTES LEARNING ABILITY DURING AGING

The insulin/IGF-1 (IIS) signal transduction pathway and its downstream effectors have been found to influence lifespan in a wide range of diverse organisms, suggesting a tightly conserved role of these mechanisms in aging. Reduction of IIS signaling promotes longevity in *Caenorhabditis elegans* and flies (Kenyon et al., 1993; Kenyon, 2010; Partridge, 2010). Whether this function is conserved in mice and humans remains unclear (Clancy et al., 2001; Tatar et al., 2001; Bluher et al., 2003; Suh et al., 2008; Bokov et al., 2011). Main components of insulin signaling in *C. elegans* are the insulin homolog INS-1, its receptor DAF-2, and the PIP3-kinase (phosphatidylinositol-triphosphate kinase) homolog AGE-1. Insulin signaling has been implicated in learning and memory, and in neuronal aging. Reduction of IIS attenuates protein aggregation and insolubility, and prevents amyloid-beta toxic effects. These processes are tightly associated with impaired nervous system function and age-related neurodegenerative diseases (Florez-McClure et al., 2007; David et al., 2010; Keowkase et al., 2010; Zhang et al., 2011; Tamura et al., 2013). In mouse models of AD, reduced IGF1 signaling protects from disease-associated neuronal loss and behavioral impairment, allthough IGF1R haploinsufficiency does not necessarily extent lifespan in mice (Cohen et al., 2009; Bokov et al., 2011).

In *C. elegans*, IIS has been shown to influence thermotaxis learning (Kodama et al., 2006) and salt chemotaxis learning (Tomioka et al., 2006). Moreover, long-lived IIS mutants show improved ability to associate temperature with food at both young and old age (Murakami, 2007). By contrast, some of these mutants are impaired, at young age, in their ability to associate NaCl with the absence of food (Vellai et al., 2006), or to intergrate sensory stimuli, such as Cu^2^^+^ and diacetyl perception, towards decision-making (Jiu et al., 2010). Moreover, long-lived *age-1* mutant animals display delayed age-related decline of isothermal tracking and locomotion. Similarly, *age-1* and *daf-2* mutants associate temperature and starvation more efficiently compared to wild type controls, while young adults of these muants show increased temperature–food association. The enhanced association capacity of *daf-2* mutants is dependent on the neuronal Ca^2^^+^-sensor NCS-1, which modulates isothermal tracking in the amphid interneurons, a key component of the thermosensory circuit (Murakami et al., 2005). AGE-1 also acts in the benzaldehyde-sensing amphid wing C (AWC) sensory neurons to direct benzaldehyde–starvation associative plasticity (Lin et al., 2010). While, mutations in the *daf-2* IIS receptor improve memory performance in *C. elegans* early in adulthood, maintaining learning ability with age, no extension in long-term memory (LTM) during aging is evident. Reduced insulin signaling does not alter neuronal plasticity but rather establishes an association more rapidly and prolongs the duration of this association early in adulthood (Kauffman et al., 2010).

Neuronal cells not only degenerate with age but the nervous system also loses the ability to regenerate after injury. Genetic experiments indicate that axon regeneration in aging *C. elegans* motor neurons is repressed by elevated IIS, which inhibits both growth cone initiation and axon growth (but not axon guidance) in aged animals. IIS impairs regeneration by blocking the function of DAF-16, a FOXO transcription factor and downstream effector of IIS. DAF-16/FOXO is necessary and sufficient to promote neuronal regeneration in a cell-autonomous manner. (Byrne et al., 2014). DAF-16 has also been shown to promote developmental neuronal migration and to affect aspects of neuronal cell morphology, such as neurite outgrowth (Christensen et al., 2011; Kennedy et al., 2013). DLK-1, a mitogen activated kinase kinase kinase (MAPKKK) that regulates presynaptic development is downregulated by IIS. DAF-16 upregulates expression of *dlk-1* in a neuron-specific manner, to promote neuronal regeneration indipendently of lifespan (Byrne et al., 2014). In addition, DAF-16 and HSF-1, the *C. elegans* heat shock transcription factor ortholog, show neuroprotective characteristics since their activation can defer the morphological and functional defects that emerge on the synapses of touch receptor neurons with physiological aging (Toth et al., 2012).

The totality of these findings suggest that in addition to extending lifespan, reduced IIS signaling also promotes learning ability with age. However, this effect is not accompanied by maintenance or extension of long term-memory during aging. Instead, IIS signaling appears to play a more significant role in the retrieval rather than acquisition of memory.

## DIETARY RESTRICTION AND LONG-TERM MEMORY

Dietary restriction (DR), a reduction in total food intake, has been shown to increase lifespan and reduce fecundity in a wide range of organisms such as yeast, nematodes, flies, and rodents (Masoro, 2005; Mair and Dillin, 2008; Piper and Bartke, 2008). Recent studies in primates indicate that DR prevents from aging-related pathologies like brain atrophy,but it is still under debate wether it extends lifespan (Colman et al., 2009; Mattison et al., 2012; Cava and Fontana, 2013; Colman et al., 2014). Little is known about the genes mediating these effects of DR. In *C. elegans*, knock-down of *mekk-3* a homolog of the mammalian mitogen-activated MEKK3-like kinase, recapitulates DR and extends lifespan. MEKK-3 deficiency leads to reprograming of fatty acid metabolism and lowering reactive oxygen species (ROS) generation, through the nuclear hormone receptor NHR-49 and DAF-22, an ortholog of human sterol carrier protein SCP2 (Chamoli et al., 2014).

The *C. elegans* feeding-defective mutant *eat-2* has been utilized as a model of DR. *eat-2* mutants ingest food poorly and, as a consequence, are long-lived. Lifespan extension by *eat-2* mutations is at least in part mediated through a *daf-16*-independent pathway (Avery, 1993; Raizen et al., 1995; Lakowski and Hekimi, 1998; Panowski et al., 2007). DR has also been suggested to attenuate age-related cognitive decline in rats (Adams et al., 2008). In *C. elegans*, young adult *eat-2* mutants show increased consistency of isothermal tracking (temperature–food association; Murakami et al., 2005). Contrary to *daf-2*, *eat-2* mutants exhibit significantly impaired LTM during young adulthood, but maintain memory capacity longer with age. Although young *eat-2* mutants display normal benzaldehyde chemotaxis, they require more training to form long-term memories. The duration of short-term memory in *eat-2* animals is similar to wild type, contrary to significant short term associative memory extension observed in *daf-2* mutants (Kauffman et al., 2010).

Dietary restriction also affects learning performance during aging in *Drosophila melanogaster*. The performance of young and old flies in an aversive learning test, where an odor is associated with a noxius mechanical shock, has been examined. These experiments showed that dietary-restricted flies, that live on average 14% longer than rich-diet fed flies, appear to have a better learning ability, even at old age. Young, dietary restricted flies show enhanced mid-term memory but their short-term memory is not affected. By contrast, short-term memory of mid-aged flies is poorer, compared with flies that grew on rich diet. Mid-term memory performance of mid-aged and old flies is not improved (Burger et al., 2010). These results are consistent with findings in *C. elegans*, in that only long term-memory is affected by DR during aging (Kauffman et al., 2010). While DR and reduced IIS signaling both increase longevity, the two pathways influence cognitive ability of young adults in an opposing manner.The differential effects of IIS and DR on learning and memory decline with age are likely due to their differential regulation of expression levels and activity of CRH-1, the cyclic adenosine monophosphate (cAMP) response element-binding protein (CREB) transcription factor homolog in *C. elegans* (Kauffman et al., 2010).

## MITOCHONDRIAL FUNCTION AND COGNITIVE AGING

Mitochondria play pivotal role in adenosine triphosphate (ATP) production, calcium homeostasis, and apoptosis regulation, and are the main source of endogenous ROS. The functionality of these organelles influences aging through multiple pathways that may be directly or indirectly relevant to cognitive decline. The link between mitochondrial dysfunction, neurodegeneration, and cognition has been a subject of intensive study in many metazoans, ranging from *C. elegans* to humans (Bishop et al., 2010; Aksenov et al., 2013). A growing body of evidence suggests that neuronal structure and function are particularly vulnerable to mitochondrial function impairment (Stein and Murphy, 2012). However, the contribution of mitochondria to selective neurodegeneration in a variety of neurodegenerative pathologies associated with cognitive decline remains a matter of debate.

Aging studies in invertebrate model organisms provide a common ground for mitopathology and cognitive research. Several conserved groups of genes influencing mitochondrial metabolism, neural plasticity and synaptic function show expression changes during aging. In *C. elegans*, loss of α-tubulin acetyltransferase gene *mec-17* causes axon degeneration, thereby leading to neuronal dysfunction. Axons lacking MEC-17 contain less mitochondria, display transport defects, and loss of synaptic integrity (Neumann and Hilliard, 2014). Atat1, the mouse homolog of MEC-17 is associated with the formation of dentate gyrus, which is essential for learning and memory (Kim et al., 2013). Moreover, studies in *C. elegans ric-7* mutants, where axonal mitochondria trafficking is impaired, suggest that mitochondria are important for protection of axons against degeneration (Rawson et al., 2014). During physiological aging, nematode touch receptor neurons display morphological and functional abnormalities, such as neurite outgrowth defects and reduced number of synapses. Positioning of mitochondria in branches required for neurite outgrowth and the accumulation of vesicles in neuronal processes suggests that trafficking deficiency underlies these age-related abnormalities (Toth et al., 2012). These morphological changes of neurons have been associated with a decline in cognition, learning, and memory during aging (Vohra et al., 2010; Kimata et al., 2012; Kim et al., 2013; Wang et al., 2013).

The nematode genome encodes five superoxide dismutases (SODs) that function in cytoplasm, mitochondria, and extracellularly. Specifically, SOD-1 regulates detoxification of syperoxide radicals in mitochondria and guards from accumulation of oxidative damage during aging (Harman, 1968; McCord and Fridovich, 1969; Yanase et al., 2009; Back et al., 2010). Nonetheless, lifespan extension in mutants overexpressing *sod-1* is not related with reduction of oxidative damage (Cabreiro et al., 2011). Transgenic *C. elegans* expressing the human G93A SOD1 variant, associated with familial amyotrophic lateral sclerosis (ALS), in motor neurons show motor defects and increased autophagy in an age-dependent manner (Aksenov et al., 2013; Li et al., 2013). SOD-1 overexpression has also been associated with mitochondrial swelling, and learning and memory impairment in flies, mice, and humans (Shin et al., 2004; Perluigi and Butterfield, 2012; Haddadi et al., 2014). For example, transgenic flies expressing a zinc-deficient SOD1 mutant display behavioral defects, including impairment of locomotion, associated with mitochondrial respiratory chain deficiency and matrix vacuolization, that is not accompanied by shortening of lifespan (Bahadorani et al., 2013). Moreover, SOD-1 activity and expression levels decline during normal aging of *Drosophila*. At the same time, knock-down of *sod-1* in the mushroom bodies deteriorates mid-term memory and LTM. These memory defects associate with reduced synapse formation and mitochondrial damage during *Drosophila* aging (Haddadi et al., 2014).

Converging evidence implicates members of the antiapoptotic BCL-2 family of proteins in neuronal injury and synapse deformation, through impairment of mitochondrial dynamics (Berman et al., 2009). CED-9, the *C. elegans* homolog of BCL-2, interacts with the mitofusin FZO-1 and the dymanin related protein EAT-3 to promote mitochondrial fusion under specific conditions. The *C. elegans eat-3* encodes a homolog of human OPA-1 which is associated with Dominant Optic Atrophy disorder (Breckenridge et al., 2009; Rolland et al., 2009). In *Drosophila*, the BCL-2 homologous proteins, Buffy and Debcl are involved in the permeabilization of mitochondria to cytochrome-*c* that is mediated by pro-death mitochondrial proteins including Reaper and Hid (Abdelwahid et al., 2011). Unlike in worms, Buffy inhibition results in normal flies, while knockdown of Debcl protects against polyglutamine (polyQ)-induced neurodegeneration through maintaining mitochondrial homeostasis. The Debcl ortholog in mice, Bax/Bak, was found to regulate neurogenesis in adult brain regions such as hippocampus and cerebellum and promote discrimination learning without affecting significantly spatial memory and learning (Senoo-Matsuda et al., 2005; Galindo et al., 2009; Sahay et al., 2011; Hardwick and Soane, 2013).

Neurons are particularly vulnerable to mitochondrial dysfunction. Interestingly, expression of the human mitochondrial uncoupling protein (UCP) *ucp2* in *Drosophila* dopaminergic neurons increases ATP production and locomotion activity, and results in neuroprotection against pathogenic stress associated with PD (Islam et al., 2012). Beyond neurons, enhanced expression of mitochondria UCPs in flies ameliorates HD phenotypes in glia cells by moderating ROS and ATP production (Besson et al., 2010). In *C. elegans*, depletion of UCP-4 exacerbates neuronal toxicity in animals expressing an expanded polyQ repeat protein in touch neurons, suggesting that similarly to flies, under normal conditions UCP-4 protects from neuronal injuries in worms (Parker et al., 2012). However, overexpression of *ucp-4* in worms does not extend lifespan (Sagi and Kim, 2012). Alterations in the expression of mitochondrial respiratory chain genes result in similar effects. For example, mutations in the *mev-1* and *gas-1* genes, encoding subunits of complex II and I of the respiratory chain, respectively, increase ROS production, shorten lifespan, and retard behavioral rates (Kayser et al., 2004). In another example, animals carrying mutations in mitochondria complex IV* sft-1* gene, show increased lifespan that is dependent on DAF-16 (Maxwell et al., 2013). Depletion of SURF1, the mouse ortholog of *sft-1*, also increases lifespan and improves cognitive function in mice (Lin et al., 2013a). Knockdown of *clk-1*, a gene required for ubiquinone biosynthesis reduces respiration rates and increases *C. elegans* lifespan, also delaying behavioral rates (Rea et al., 2007). Loss-of-function mutations in *clk-1* extend lifespan and slow development and behavioral rates (Takahashi et al., 2012). Similarly, knockdown of the mouse *clk-1* ortholog causes mild mitochondrial dysfunction and extends lifespan (Lapointe and Hekimi, 2008; Deepa et al., 2013). In *Drosophila,* reduced expression of complex I and IV genes specifically in adult neurons is sufficient to extend lifespan (Copeland et al., 2009). Furthermore, observations in *clk-1* mutants indicate that neurite outgrowth is inhibited in aged worms (Tank et al., 2011). The association between neuronal morphology and behavioral effects suggests that mitochondria dysfunction may, in part, underlie memory and learning decline during aging (Ardiel and Rankin, 2010; Kimata et al., 2012; Stein and Murphy, 2012). However, little is known about the molecular mechanisms that mediate the effects of alterations in mitochondrial metabolism on both cognitive capacity and longevity.

## AUTOPHAGY AND PROTEIN HOMEOSTASIS IN LEARNING AND MEMORY

The autophagic pathway has also been implicated in aging and cognitive decline. Autophagic activity decreases during the course of aging and genes that control this process are strongly associated with lifespan regulation in flies and worms (Lionaki et al., 2013). In *Drosophila*, overexpression of autophagy-related genes in neurons enhances longevity, while their repression causes neuronal defects and shortening of lifespan (Simonsen et al., 2008). Similarly, increasing autophagy mediates lifespan extension in worms (Hansen et al., 2008). UNC-51, a nematode autophagy regulator also controls axonal and dendritic development and its homolog affects olfactory learning in flies (Mochizuki et al., 2011). Worms lacking UNC-51 display axonal membrane defects, indicating a role of autophagy in synaptic plasticity, which indirectly interferes with learning and memory (Sigmond et al., 2008; Ragagnin et al., 2013). In *Drosophila*, inhibition of the cyclin-dependent kinase 5 (cdk5) kinase ortholog decreases autophagy, shortens lifespan and causes structural defects in central brain regions associated with olfactory learning and memory (Trunova and Giniger, 2012). In both flies and worms, autophagy deficiency leads to abnormal accumulation of protein aggregates thus promoting pathological mechanisms associated with neurodegenerative disorders, such as HD and AD (Ling et al., 2009; Low et al., 2013). For example, accumulation of intracellular APL-1, a β-amyloid precursor protein, upon autophagy impairment, causes behavioral deficiencies, including olfactory and gustatory learning defects, and habituation delay in *C. elegans* (Ewald et al., 2012; Ewald and Li, 2012; Chen et al., 2013). Accumulation of APL-1 also occurs during normal aging and can reach pathological levels contributing to the pathogenesis of AD (Nilsson et al., 2013).

Age-induced memory impairment studies in *Drosophila* suggest that cognitive aging is strongly associated with the autophagic pathway. Indeed, spermidine-induced autophagy reduces aggregation of ubiquitinated proteins and protects from age-related memory impairment, in the aged *Drosophila* brain (Gupta et al., 2013). Spermidine activates autophagy to also promote longevity in different metazoans ranging from *C. elegans* to mice (Eisenberg et al., 2009; Wang et al., 2012). Other studies suggest that spermidine may not act directly through autophagy to facilitate neuroprotection and memory during aging. Instead, spermidine administration may influence histone acetyltransferase activity to modulate autophagy (Simonsen and Tooze, 2009; Davis, 2013; Graff and Tsai, 2013). These findings indicate that although the protective effect of spermidine does require activation of the autophagy pathway, the involvement of additional regulatory pathways remains to be elucidated. In conclusion, the exact mechanism by which autophagy controls cognitive aging is multifaceted and remains poorly understood. Additional studies are required to elucidate the contribution of autophagy in both longevity and cognitive capacity maintenance during aging.

## TOR SIGNALING AND LONG-TERM MEMORY

Reduced signaling through the target of rapamycin (TOR) kinase has been shown to extend lifespan in diverse organisms (Vellai et al., 2003; Jia et al., 2004; Kapahi et al., 2004; Kaeberlein et al., 2005; Powers et al., 2006; Kenyon, 2010). The evolutionarily conserved mTOR functions in two complexes, mTORC1 and mTORC2 (Hay and Sonenberg, 2004; Wullschleger et al., 2006; Guertin and Sabatini, 2007). Tight regulation of the upstream components of the TOR pathway is important for proper neural growth and function throughout development and adulthood in *C. elegans* (Goldsmith et al., 2010). Overexpression of the small GTPase RAS homolog enriched in brain (Rheb), an upstream activator of TOR, in *Drosophila* photoreceptor cells downregulates autophagy, causes axon guidance defects and induces cell death (Knox et al., 2007; Wang et al., 2009). Selective overexpression of Rheb in distinct subsets of central brain neurons results in enlarged cell bodies and projections. In addition, Rheb overexpression in the mushroom bodies decreases mid-term odor-sucrose memory (Brown et al., 2012).

In the PIP3/PTEN/Akt/TOR pathway phosphorylated Akt activates TOR to regulate cell cycle and protein synthesis. In flies, the PTEN/Akt pathway is implicated in axon regeneration (Song et al., 2012). Similarly, axon regeneration is evident after the loss of DAF-18/PTEN in young adult worms (Byrne et al., 2014). Reduced TORC2 activity causes LTM deterioration in fruit flies (Huang et al., 2013). Rapamycin, a protein synthesis inhibitor that acts through the TOR pathway (mainly mTORC1), blocks long-term facilitation (LTF) in *Aplysia californica* (Hu et al., 2006). Moreover, rapamycin completely disrupts pre-existing long-term synaptic plasticity in *Aplysia* (Hu et al., 2011). While rapamycin extends lifespan mainly by blocking the TOR pathway, it may exert its effects on cognition through a different mechanism (Neff et al., 2013).

The TOR pathway controls translation of 5′TOP mRNAs containing a 5′ terminal oligopyrimidine tract. 5′TOP mRNAs encode proteins of the translational machinery. Under physiological conditions, 5′TOP mRNAs are largely repressed. Serotonin, which activates the TOR pathway, aleviates this repression, in a rapamycin-sensitive manner (Garelick and Kennedy, 2011; Labban and Sossin, 2011). eEF2 (eukaryotic elongation factor 2) is implicated in LTF in *Aplysia*, but is differentially regulated by eEF2 kinase in the neurites and the soma of sensory neurons involved in LTF (Weatherill et al., 2011). TORC1 mediates regulation of phosphorylation of eEF2 through the eEF2K (Carroll et al., 2004). Both in *Aplysia*, and in rodents, eEF2K function is associated with increased memory processing, through enhancing expression of genes implicated in the regulation of syntaptic strength (Weatherill et al., 2011).

Similarly, long term administration of rapamycin eliminates neuronal demyelination and neurodegeneration observed during aging in senescence-accelerated OXYS rats, a strain characterized by overproduction of free radicals (Kolosova et al., 2013). In mouse models of AD, rapamycin administrated either prior or after the onset of AD symptoms, improves animal cognition, probably through the preservation of brain vascular integrity and function (Lin et al., 2013b). Moreover, chronic treatment with rapamycin enhances spatial learning and memory with age, as well as the ability to recall a memory, even when the administration takes place late in life (Halloran et al., 2012). However, short-term administration following the emergence of learning and memory defects with aging, is not accompanied by such positive effects. The improvement of cognitive ability with rapamycin is mediated through reduction of TOR signaling and of IL-1β levels in the hippocampus, the facilitation of NMDA signaling, and increased CREB phosphorylation (Majumder et al., 2012). Furthermore, increased phosphorylation of S6, a target of TOR, is observed in the prefrontal cortex, after the administration of rapamycin, in OXYS rats (Kolosova et al., 2013).

## CONCLUSION

**FIGURE 1:**
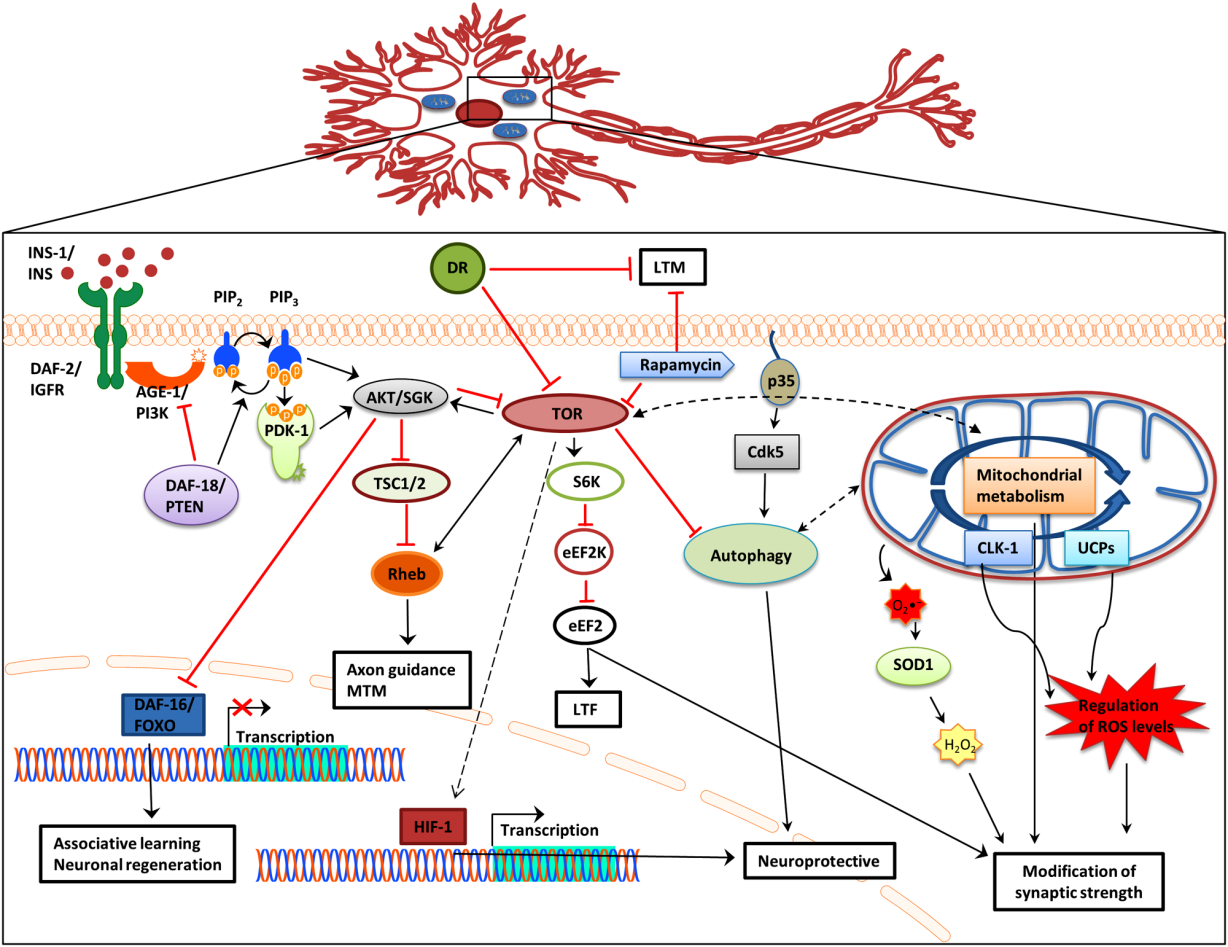
**Effects of IIS, DR, and TOR signaling, autophagy, and mitochondrial function on memory formation.** In addition to extending lifespan, attenuation of IIS signaling, and subsequent de-repression of DAF-16 also reinforces associative learning and promotes axon regeneration and neuronal migration. Dietary restriction significantly impairs long-term memory, while it does not affect short-term memory. Blocking TOR signaling causes long-term-facilitation defects, while Rheb overexpression decreases mid-term memory and causes axon guidance defects. Regulation of autophagy through Cdk-5 affects lifespan and has also been associated with central nervous system structure defects, including olfactory learning and memory. Several mitochondrial genes promote neuroprotection. Impairment of mitochondrial function causes over production of ROS and concomitant synaptic deficiency. AGE-1/PI3K,phosphoinositide 3-kinase; AKT, protein kinase B; Cdk5, cyclin-dependent kinase 5; CLK-1, clock 1; DAF-2/IGFR, insulin-like growth factor receptor; DAF-16/FOXO, forkhead box O; DAF-18/PTEN, phosphatase and ensin homolog; DR, dietary restriction; eEF2, eukaryotic elongation factor 2; eEF2K, eukaryotic elongation factor 2 kinase; HIF-1, hypoxia-induced factor 1; INS, insulin; LTF, long-term facilitation; LTM, long-term memory; MTM, mid-term memory; PDK-1, 3-phosphoinositide-dependent kinase 1; PIP_2_, phosphatidylinositol (4,5)-bisphosphate; PIP_3_, phosphatidylinositol (3,4,5)-trisphosphate; Rheb, Ras homolog enriched in brain; S6K, ribosomal protein S6 kinase; SGK, serum- and glucocorticoid-inducible kinase; SOD1, superoxide dismutase 1; TSC1/2, tuberous sclerosis 1/2; TOR, target of rapamycin; UCPs, uncoupling proteins; Black arrow, direct stimulation; black dashed arrow, indirect stimulation; black dashed double head arrow, interplay; red arrow, inhibition.

Understanding how neuronal aging and cognitive impairment are influenced by mechanisms that modulate lifespan is an ongoing challenge. Such well-studied mechanisms include the IIS signaling pathway, DR, mitochondrial dysfunction, autophagy, and the TOR signaling pathway. Accumulating evidence indicates that these pathways also impinge on age-related neuronal dysfunction and memory-impairment. Indeed, manipulation of these pathways in a variety of metazoans affects neuronal structure and function and consequently promotes age-related memory impairment. It is likely that the decline in different forms of memory is independently mediated by distinct aging mechanisms (**Figure 1**). Decreased IIS signaling promotes decision making and associative learning. However this is not a general rule and, instead, appears to be dependent on different types of association. Nonetheless, DAF-16 activation likely delays morphological changes that occur with aging and promotes neuronal regeneration. DR exerts negative effects on LTM but enhances association making and memory. DR effects on short-term and mid-term memory appear to be age dependent.

The TOR pathway influences cognition by controlling protein biosynthesis, cell cycle, and metabolism (Garelick and Kennedy, 2011; Santos et al., 2011). Studies in invertebrates suggest that increased TOR signaling downregulates autophagy and causes axon guidance defects, while also promoting memory processing and synapse integrity. Conversely, in addition to increasing lifespan, downregulation of TOR signaling, mainly through rapamycin treatment, blocks LTM and LTF but also causes axon regeneration. In rodents rapamycin administration offsets the negative impact of aging on spatial learning and memory, increases memory recall ability, and enhances the vascular integrity of the brain. Moreover, morphological signs of AD and aging, such as neuronal demyelination and neurodegeneration are ameliorated. Activation of mTORC1 promotes mRNA translation, which likely enhances synapse formation. These distinct, but not necessarily conflicting results of manipulating TOR signaling could reflect a dose or compartment dependent regulation of cognition through TOR signaling.

In both worms and flies, activation of autophagy appears to promote lifespan and cognitive function. In mammals, little it is known about the role of autophagy during aging. However, recent studies in mice suggest that overexpression of autophagy-related gene Atg5 also increases lifespan (Pyo et al., 2013). Manipulation of mitochondria function during aging causes similar effects, either causing axon degeneration, or promoting axon integrity and stability (Keller et al., 2011; Fang et al., 2012). These ostensibly contradictory observations may suggest a neuron-specific function of mitochondria in aging. Despite recent progress and findings, several open questions need to be addressed. The involvement of epigenetic mechanisms and environmental conditions on nervous system aging is largely unknown. In addition, whether aging differentially affects subpopulations of neurons or different brain areas and to what extent remains unclear. While significant progress has been achieved towards deciphering the link between pathways that modulate both lifespan and aspects of neuronal function in invertebrate models, the relevance of these findings to neuronal aging and pathophysiology in higher organisms including humans has not been evaluated yet. Addressing these key issues will contribute towards developing informed strategies and therapeutic approaches aiming to battle age-related decline of nervous system performance and numerous neurodegenerative conditions associated with aging.

## Conflict of Interest Statement

The authors declare that the research was conducted in the absence of any commercial or financial relationships that could be construed as a potential conflict of interest.
